# Burden of neurological illnesses in a pediatric intensive care unit of developing country

**DOI:** 10.12669/pjms.306.5671

**Published:** 2014

**Authors:** Qalab Abbas, Amber Shabbir, Naveedur Rehman Siddiqui, Raman Kumar, Anwarul Haque

**Affiliations:** 1 Qalab Abbas, MBBS, Department of Pediatrics and Child Health, Aga Khan University Hospital, Karachi, Pakistan.; 2 Amber Shabbir, MBBS, Department of Pediatrics and Child Health, Aga Khan University Hospital, Karachi, Pakistan.; 3 Naveedur Rehman Siddiqui, FCPS, Department of Pediatrics and Child Health, Aga Khan University Hospital, Karachi, Pakistan.; 4 Raman Kumar, MBBS, Department of Pediatrics and Child Health, Aga Khan University Hospital, Karachi, Pakistan.; 5 Anwarul Haque, MBBS, Department of Pediatrics and Child Health, Aga Khan University Hospital, Karachi, Pakistan.

**Keywords:** Burden, Acute Neurological illnesses, PICU, Mortality

## Abstract

***Objective:*** To assess the burden and spectrum of neurological illness in a pediatric intensive care unit and review the associated mortality.

***Methods:*** Retrospective review of medical records of children (1 mo-16 years) with acute neurological diagnosis admitted in PICU in Aga Khan University hospital from January 2008 to December 2011 was done. Basic demographic, diagnosis, neuro diagnostic procedures performed, therapies and outcomes were done on a structured datasheet.

***Results:*** During study period, 231 (19.3%) patients were admitted with acute neurological illnesses in PICU. The mean age was 67 ±50 months, 54% (n=125) was under-five and 138 (59.7%) were males. Out of total, 144 (62.3%) had neurological illness and 87 (37.7%) had neurosurgical diagnosis. In acute neurological illness, 51.5% (n=119) had non-traumatic-coma (NTC) and 10.8% (n=25) had neuromuscular illness. CNS infection (26%, n=60) in structural cause and status epilepticus (10%, n=23) were the most common cause of structural and metabolic type of NTC respectively. Severe traumatic brain injury (21.2%, n=49) and postoperative neurosurgical illness (16.5%, n=38) were common neurosurgical cases in our cohort. The intensive care resources were utilized as mechanical ventilation in 78% (n=180), inotropic support in 29.4% (n=67) and therapeutic hypothermia in 33% (n=76). Fifty children (21.6%) required PICU care for observation only. More than 500 neurodiagnostic tests/procedures were performed in this cohort of children with acute neurological disorders in PICU. The mortality rate in neurological cases was 18% (42/231) as compared to the overall mortality rate was 12% in PICU.

***Conclusion: ***Acute neurological disorders were common in PICU, and were associated with higher mortality. CNS infections, status epilepticus and severe traumatic brain injuries were the most common acute neurological illnesses in our cohort.

## INTRODUCTION

The burden of neurological illnesses is high in children. Acute neurological illnesses constitute about one-third of emergency department visits and 25% of pediatric intensive care unit admissions.^1^ Both primary neurological illnesses and neurological complications of systemic illness are commonly encountered in pediatric intensive care unit (PICU).^2^^,^^3^ PICU patients with neurological injuries have higher chances of mortality, morbidity and longer length of hospital stay.

 A recent study found that more than half of the patients who died in a tertiary care center PICU had an acute brain injury.^4^ Along with treatment of primary illness, prevention and treatment of secondary injuries are very critical. Several studies have demonstrated that the hypoxia and hypotension are the major cause of secondary injuries. Implication of neuro-resuscitation and neuroprotective strategies as well as advances in diagnosis and therapeutic armamentariums has significantly improves the outcome of various neurological illnesses.^5^^,^^6^ Few acute neurological disease-specific incidence and outcome in children from Pakistan have been published.^7^^,^^8^ A comprehensive review of burden of acute neurological illness in children is lacking. The objective of this study was to assess the burden and review the spectrum of acute neurological cases which were admitted in our pediatric intensive care unit.

## METHODS

Medical record of children from age 1 month to 16 years, admitted in our pediatric intensive care unit with the primary diagnosis of a neurological illness, from January 2008 to December 2011, were reviewed retrospectively. The study was approved by the ethical review committee of Aga Khan University (2366-PED-ERC-12). Aga Khan University Hospital (AKUH) is a tertiary-care, teaching hospital with several pediatric sub-specialties in the department of pediatrics including pediatric critical care medicine and pediatric neurologist who were trained from accredited fellowship and board certified in their discipline.

 Our PICU is a multidisciplinary-cardiothoracic closed ICU with average 450 annual admissions. Any illness which primarily involved the nervous system on admission was labeled as a neurological illness. Diagnosis was divided into two main categories Neurological diagnosis and Neurosurgical diagnosis (ICD 9 coding system). Neurological category was further subdivided into Non-traumatic Coma (NTC) and Neuromuscular disorders (NMD). NTC was divided into structural/intrinsic (CNS infections, vascular and space occupying lesion) and metabolic/toxic (encephalopathy due to hypoxia, hypertension, status epilepticus, poisoning and metabolic like inborn error of metabolism, diabetic coma, fulminant hepatic failure). Neuromuscular diagnoses were mainly Guillain-Barre syndrome (GBS), Myasthenia Gravis, and botulism etc. Neurosurgical cases were included traumatic brain injury and postoperative care after any neurosurgical procedures.CNS infections were mainly meningitis, encephalitis and cerebral malaria diagnosed on the basis of clinical examination, results of lumbar puncture and brain imaging (CT scan and or MRI brain). Encephalopathy was subdivided into hypoxic/ischemic, metabolic, toxic or status epilepticus. All diagnosis were made on basis of history, physical examination, laboratory tests, neurodiagnostic tests like imaging CT/MRI, EEG/NCV/EMG and therapeutic interventions like therapeutic hypothermia and plasma exchange were done according to the condition of the patient and suspected diagnosis as per pediatric intensivist and pediatric neurologist.

The following data was collected on structured propforma which included *basic demographic* (age, gender, primary diagnosis),*ICU resource utilization*- use of mechanical ventilation, inotropic support, therapeutic hypothermia and plasmapheresis and *outcome variables* as survival/expired, length of stay and Glasgow coma outcome scale(GCOS). GCOS was divided into two group based on prognosis: Good (4-5) and Bad (<3). The neurodiagnostic procedures like EEG, EM/NVC, SSEP, and MRI/CT of brain, radionuclide cerebral perfusion scan and lumbar punctures were also recorded. Descriptive statistics and appropriate analytical tests were applied. 

## RESULTS

**Fig.1:**
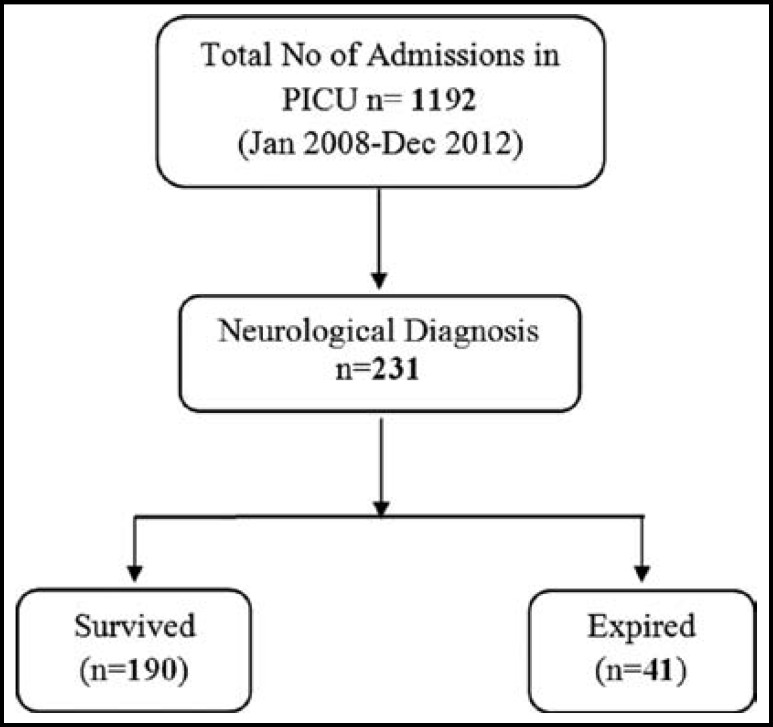
Flow Diagram of study.

During the four year study period, a total of 1192 patients were admitted in our PICU as shown in Fig.1. Mean age was 67±50 months, 59.7% were males. A total of 231 (19.37%) patients were diagnosed as having a neurological diagnosis. The frequency and percentage of different diagnostic categories is shown in Table-I. Of total, 144 (62.3%) has neurological diagnosis and 87(37.7%) had neurosurgical diagnosis. In neurological group, NTC cases were 51.5% (119) and neuromuscular cases were 10.8% (n=25). The intrinsic/structural group of NTC comprised of 32.4% of total cases (n=75) and included 26% (n=60) CNS infections, 4.1% (n=10) and 2% (n=5) cases of brain atrophy. The metabolic/toxic type of NTC was 19% of total (n=44), which included status epilepticus 10% (n=23), hypoxic-ischemic encephalopathy 3.4% (n=8) and metabolic/idiopathicencephalopathies 5.6% (n=13). The neuromuscular disorders consisted of 10.8% (n=25) which included Guillain-Barre syndrome (n=14), Myasthenia Gravis (n=10) and botulism (n=1). Neurosurgical disorders included severe traumatic brain injury 21.2% (n=49) and postoperative care after neurosurgical procedures 16.5% (n=38).

The intensive care resources were used in 78.3% of patients (n=181) while fifty cases (21.6%) of acute neurological disorders were admitted only for observation in our PICU. Mechanical ventilation was required in 78% of the patients (33.7%), 29.4% (n=67) required inotropic support while in PICU, and 33.3% (n=76) received therapeutic hypothermia for neuroprotection. Therapeutic plasma exchange was done for 13 patients in neuromuscular illness. CT scan brain was done in 70% (n=162) of these patients, MRI in 42% (n=97), LP in 33% (n=76), radionuclide cerebral perfusion scan in 3.5% (n=8), EEG in 65% (n=150), continuous EEG in 10% (n=23) and somatosensory evoke potential (SSEP) for prognosis of coma in 3.5% (n=8) as shown in Table-II.

The mean length of PICU stay was 5.68±6.47 days. 17.7% (n=41) of patients had Glasgow coma outcome scale of <3 while 18.6% (n=43) patients died in acute brain injury group as compared to 12% (n=143) in overall mortality.

Patients who received mechanical ventilation and inotropic agents had higher chance of dying as compared to who did not (*p<0.001*) on multivariate analysis.

## DISCUSSION

To the best of our knowledge, this is the first comprehensive report which describes the entire spectrum of acute severe neurological and neurosurgical illness in children from tertiary-care multidisciplinary PICU of Pakistan. We found that 19.3% (231/1192) of all PICU admissions were categorized as acute neurological insults. Few studies have been published on the CNS-specific acute illness like Status Epilepticus, CNS-infections, Stroke, Nontraumatic coma and Traumatic Brain Injuries in children from Pakistan.^7^^-^^9^ The spectrum of neurological disease has very wide range. The burden of neurological illnesses among the seriously acutely sick children is very high in developing countries like Pakistan. Few studies have shown that the majority of deaths in PICU were associated with neurologic failure.^4^

**Abbreviations** (*PICU *pediatric intensive care unit).

**Table-I:** Demographic, Resource Utilizations, and Outcome variables of critically ill children admitted with a Neurological diagnosis in Pediatric Intensive Care Unit (n-231).

**Variables**	**N= 231 (100%)**
Age (mo) (mean ± SD)	67 ±50
Male	138 (59.7)
**ICU Interventions**	
MV	181(78.4)
Inotropes	68 (29.4)
Therapeutic Hypothermia	77 (33.3)
Plasmapheresis	13 (5.6)
**Neurodiagnostic Procedures**	
CT scan Brain	162 (70)
MRI	97 (42)
LP	76(33)
EEG	150(65)
cEEG	23 (10)
SSEP	8 (3.5)
Nuclear Perfusion Scan	8 (3.5)
**Outcome Variables:**	
Length of ICU stay (days) (mean ± SD)	5.68 ± 6.47
Glasgow Coma outcome scale >3	41 (17.7)
Expired	42 (18.6)

**Table-II:** Frequency of Acute Neurological and Neurosurgical disorders and mortalities

**Variables**	**Cases n (%)**	**Mortality n (%)**
All Neuro-neurosurgical Cases	231 (100)	42 (18.18)
Neurological Cases	144(62.3)	24 (16.6)
Neurosurgical Cases	87(37.7)	18 (26.6)
**Neurological Cases**	144(62.3)	24 (16.6)
Non-traumatic Coma	119 (51.5)	21 (9)
*Structural*	75 (32.4)	13 (17.3)
CNS Infection	60 (26)	11(18.33)
Stroke	10 (4.32)	1(10)
Degenerative disease	5 (2.16)	1
*Metabolic / Toxic*	44 (19.0)	8 (18.18)
Status Epilepticus	23 (10)	1 (4.34)
Hypoxic-Ischemic	8 (3.4)	4
Other metabolic	13(5.62)	2
Neuromuscular Disease	25 (10.8)	3 (12)
GBS	14	0
MG	10	3 (30)
Botulism	1	0
**Neurosurgical Cases**	87 (37.7)	18 (20.2)
Traumatic Brain Injury	49(16.5)	8 (16.3)
Post-operative NS:	38 (21.2)	10 (26.31)

Our study shows that almost one-fifth of all admissions in PICU was acute neurological illnesses and was associated with comparatively higher mortality rates like other previous published reports. Bell et al. 26.2% acutely ill child were admitted to PICU with neurological diagnosis.^2^ Similarly, LaRovere et al. reported one in five PICU admissions had diagnosis of acute neurological illness in their cohort.^3^ Children with neurological diagnosis in PICU had a longer hospital length of stay and greater hospital cost.^1^ Recently, Elbeleidy et al. described pattern of neurological disorders in their PICU and constitute 30% of the total PICU admissions.^10^

Several studies have reported that CNS infections are the major cause of acute neurological illness and non-traumatic coma in children from developing countries, including meningitis, encephalitis and cerebral malaria.^8^^,^^11^ On the other hand, the rates of CNS infections in PICU of developed countries were low.^2^ Infections of CNS was 26% (n=60) in our cohort. Other reports from developing countries showed that CNS related infections constitute about 50% of all acute neurological disorders. Acute seizure, status epilepticus and traumatic brain injuries were the most common reasons for PICU admissions in USA.^1^^-^^3^ Status Epilepticus and Traumatic Brain Injury accounted for 10% (n=23) and 16.5% (n=49) of neurological cases in our PICU respectively.

The mortality rate was 18.18% (n=42) in our cohort is high as compared to the overall mortality in our PICU (12%). The use of mechanical ventilation and inotropes in children with acute neurological disorders were associated with higher mortality rate (*p*<0.001). There has been a wide range of mortality in acutely ill or injured children with neurological or neurosurgical disorders in different PICUs across the globe (5 % - 60%).^2^^,^^8^^-^^11^ There is a significant difference in pattern of mortality from neurological illness between developed and developing countries. All three reports of pediatric neurocritical from USA have demonstrated that admission rate was 20-30% of all PICU admissions and the mortality rate was only 4-7%.^1^^,^^2^^,^^12^ The mortality rate in acute neurological illness in children from developing countries was reported from 30-60% in several studies.^11^^,^^13^^,^^14^ Our cohort also have higher mortality rate (18.18%). This high mortality is attributed to several factors, including late presentation to hospital, severity of neurological illness and inadequate resources for intensive care needs. The care of acutely ill child with neurological illnesses is complex and requires careful balancing of cerebral and systemic priorities. Adult neurocritical care is a well-established discipline in developed countries, which has shown improvement in the outcome and shorter length of stay.^15^^-^^17^ Recently, the pediatric neurocritical care is evolving as a subspecialty in west. It has been emphasized that early recognition of illness and timely appropriate interventions can significantly improve the outcome.^18^^-^^20^ We also have published that there was a significant improvement in outcome of status epilepticus and severe traumatic brain injuries before and after implementation of pediatric neurocritical care service.^13^^,^^21^ The most of acute neurological disorders in children have a high potential of full recovery if it is recognized early and initiate timely appropriate interventions.^22^ Like Emergency Neurological Life Support (ENLS) course, we have initiated an educational program “Pediatric Neurologic Emergency Life Support” (PNELS) course with intention to standardize the care of acutely ill child with common neurological disorders with emphasis on early recognition and a set of time-sensitive, goal-directed protocol for treatment since November 2013.^23^ This course emphasized heavily on neuroresuscitation and neuroprotective strategies to prevent and treat neurological and systemic secondary injuries which have devastating consequences on neurological morbidity and mortality. The objective of this course is that participants will be able recognizing quickly and rapidly initiate protocol-based management in children with acute life-threatening neurological and neurosurgical disorders. Several evidences are available that adherence to protocols used in neurocritical care improve outcomes.^16^^,^^20^ The aim of this course is to standardize the care with particular do’s and don’ts in the recognition and management of acutely child with neurological emergencies for all health care providers a in the arena of severe shortage of trained pediatric neurologist and pediatric intensivist.

There are several limitations to our study. Data was collected retrospectively from a single center. We didn’t assess the severity of illness like PRISM III score. We didn’t include encephalopathy secondary to complicated diabetic ketoacidosis, septic encephalopathy and fulminant hepatic failure. The strength of this study is the first comprehensive report on spectrum of acute neurological and neurosurgical illnesses from Pakistan.

## CONCLUSION

Acute neurological disorders are common in PICU, and are associated with higher mortality. CNS infections, status epilepticus and severe traumatic brain injuries were the most common neurological illnesses in our cohort.

## Author’s Contribution:

**AH:** Concept, Design, and final guarantor. **QA: **writing manuscript.

**RK and AS: **Data collection. **NRS:** Statistical Analysis.

## Notes

***Funding: ***None.
